# Children with an Autism Spectrum Disorder and Their Caregivers: Capturing Health-Related and Care-Related Quality of Life

**DOI:** 10.1007/s10803-019-04249-w

**Published:** 2019-10-17

**Authors:** Leontine W. ten Hoopen, Pieter F. A. de Nijs, Jorieke Duvekot, Kirstin Greaves-Lord, Manon H. J. Hillegers, Werner B. F. Brouwer, Leona Hakkaart-van Roijen

**Affiliations:** 1 Department of Child and Adolescent Psychiatry/Psychology, Erasmus MC, Sophia Children’s Hospital, Rotterdam, P.O. Box 2040, 3000 CA Rotterdam, The Netherlands; 2 Erasmus School of Health Policy & Management, Erasmus University Rotterdam, P.O. Box 1738, 3000 DR Rotterdam, The Netherlands; 3 Yulius Mental Health, P.O. Box 753, 3300 AT Dordrecht, The Netherlands

**Keywords:** Autism spectrum disorder (ASD), Caregiver burden, CarerQol, Children, EuroQol five-dimensional (EQ-5D) questionnaire, Health-related quality of life

## Abstract

This study investigated health-related QoL (HRQoL) and care-related quality of life (CarerQol) in clinically referred children with an autism spectrum disorder (ASD), and their primary and secondary caregivers. The EuroQol five-dimensional (EQ-5D) and the CarerQol questionnaires were used to respectively measure health-related QoL and care-related QoL. Primary caregivers reported pain/discomfort (42%) and anxiety/depression (40%). In caring, they mostly experienced problems in the relationship with the child (84%), and in combining care with daily activities (51%). Children with ASD had a relevantly lower QoL. Despite negative effects, almost all caregivers (96%) derived fulfillment from caring for their affected children. HRQoL and CarerQol reports of primary caregivers and children were correlated, both providing useful information to ASD measurement and treatment.

Children with an autism spectrum disorder (ASD) experience reduced quality of life (QoL; Bastiaansen et al. 2004; Kuhlthau et al. 2010). Compared to typically developing or chronically ill children, significantly lower QoL for children with an ASD was reported, especially regarding their psychosocial and emotional functioning (De Vries and Geurts 2015; Kuhlthau et al. 2013, 2018). Gurney et al. (2006) found that in general the children with an ASD experience more health problems than typically developing children. The multiple developmental, health, interactional, and behavioral difficulties associated with an ASD are not only challenging for the affected children in growing up but also for their caregivers in parenting (Volkmar et al. 2014). Caregivers (often parents) of children with an ASD have been shown to have an elevated risk of experiencing physical health problems and mental health problems, such as stress, anxiety, and depression, compared to caregivers of children without an ASD or the general population (Allik et al. 2006; Eapen and Guan 2016; Falk et al. 2014; Khanna et al. 2013b). Also, many of them face financial stress in caring, for example, because of high health care expenditures and reduced employment (Hoefman et al. 2014a; Kuhlthau et al. 2010). Lower QoL was found in caregivers of children with an ASD, compared to the general population (Khanna et al. 2011; Kuhlthau et al. 2014), and to caregivers of healthy, chronically ill or disabled children (Mugno et al. 2007; Kheir et al. 2012). This finding reflects not only the caregiving burden associated with parenting a child with an ASD but also has possible secondary effects on the child and vice versa. From clinical care and transactional models, we know that there is a continuous reciprocal interaction process between children and caregivers (Rodriguez et al. 2019). Thus, problems of the child with an ASD may impact the caregiver’s QoL, which in turn can influence the caregiver’s interaction with the child, which can further influence the child’s QoL. Because of this process, and the essential involvement of caregivers in treatment (Volkmar et al. 2014), measuring QoL of both children with an ASD and their caregivers is essential in outcome and treatment evaluation.

For measuring QoL in children with an ASD and their caregivers, mostly *health*-*related* QoL (HRQoL) questionnaires are used because of the presence of a health condition. These questionnaires focus on the subjective evaluation of physical, psychological, and social functioning, excluding non-health-related aspects (Ferrans et al. 2005). A generic HRQoL measure typically consists of a descriptive system, which describes the health status of the respondent (e.g., Karimi and Brazier 2016). This descriptive part of the measure often covers several health domains, for which respondents can indicate their level of functioning. The health descriptions can be linked to empirical valuations of the general public, allowing to compute utilities. Each health state has a single utility score, so-called ‘tariffs’ (Versteegh and Brouwer 2016). For example, Khanna et al. (2013b) and Kuhlthau et al. (2014) used the EuroQoL Five Domain Health Questionnaire (EQ-5D; Brooks 1996; The EuroQol Group 1990; www.euroqol.org) to evaluate the HRQoL in caregivers of children with an ASD in the United States (US). The EQ-5D generates a single utility score from the health state description based on the level of functioning in five domains (mobility, self-care, usual activities, pain/discomfort, and anxiety/depression) as an expression of HRQoL (Drummond 2001). These scores are anchored on the state dead (0) and perfect health (1), with the possibility of having scores below 0. In caregivers of children with an ASD in the US, a mean EQ-5D utility score of 0.82 was found by Khanna et al. (2013b) and 0.85 by Kuhlthau et al. (2014). The advantage of this *utility*-*based* HRQoL is the possibility to compare and evaluate across populations, disorders, and interventions (The EuroQol Group 1990; Khanna et al. 2013a).

A drawback of the HRQoL is missing out on specific caring aspects in QoL measurement. Especially in caring for children with an ASD, relational problems with the child, but also social problems or problems in combining the caring with other tasks may impact the QoL of caregivers (Karst and Van Hecke 2012). These aspects are included in care-related QoL questionnaires (CarerQol; Brouwer et al. 2009). Although studies investigating the impact of caregiving are often focused on negative effects, such as the burden and the health problems (Allik et al. 2006; Khanna et al. 2013b), caring for a child may also have positive consequences, such as fulfillment related to the caring (Brouwer et al. 2006; Hoefman et al. 2014b). The CarerQol instrument (Brouwer et al. 2006) is specially developed to measure negatively and positively perceived aspects of informal caring in different situations or disorders. In the US study by Hoefman et al. (2014a), caregivers of children with an ASD reported problems combining care tasks with own daily tasks (60.7%), mental health problems (58.1%), physical health problems (55.6%), financial problems (52.3%), and relational problems with the child (44.9%) because of the caring, but also fulfillment (97.2%) and support (76.4%) on the CarerQoL. Caregivers reported a mean happiness score of 7.4.

Until now, ASD studies typically focused on the QoL of the affected children (Kuhlthau et al. 2010) *or* the caregiver most involved in caring (i.e., primary caregiver, Hoefman et al. 2014a, b; Khanna et al. 2013b; Kuhlthau et al. 2014). In the majority of these studies, the primary caregiver was the (biological) mother of the child. Only a few studies included mothers *and* fathers in comparing their wellbeing and exploring associated factors (Allik et al. 2006; Mugno et al. 2007). In these studies, the mothers’ wellbeing was more impaired than that of fathers’. In clinical practice, we observe continuous reciprocal interactions between children and all involved caregivers, mostly the (biological) parents. Caregivers can support, reinforce, complement, but also undermine each other in their functioning. Because of the importance of the earlier discussed family transactions in parenting a child with an ASD (Rodriguez et al. 2019), the current study explored HRQoL of the children and *both* most involved caregivers, if available. Therefore, the HRQoL of the primary caregiver and the secondary caregiver were both included in this study. With the secondary caregiver, we here mean the second-most involved in caring for the child according to the primary caregiver. This caregiver might, for example, be the other parent or the partner of the primary caregiver. To take a broader perspective on the QoL of the primary caregiver, also care-related QoL was explored, since more than only health may be affected by caring for a child with an ASD.

This study aimed (1) to provide insight in the HRQoL of children with an ASD, their primary and secondary caregivers, (2) to assess negative and positive impacts of the caring on care-related QoL of the caregivers most involved in caring, i.e., the primary caregivers, and (3) to explore the possible relationship between the HRQoL of the children and caregivers with the care-related QoL of the primary caregivers. Utility-based QoL instruments were used to be able to evaluate the HRQoL and care-related QoL impact of ASD compared to other conditions. We expected the impact of ASD on the QoL of the children, primary and secondary caregivers to be related to each other but also expected each to provide unique information and possible intervention targets.

## Methods

### Study Sample and Assessment Procedure

We collected data as part of the “Social Spectrum Study”, a prospective, multicenter study of relations between autistic traits and individual, familial, and care characteristics of clinically referred children and their families (Duvekot et al. 2017). All children aged 2–10 years referred to one of six child and adolescent mental health services (CAMHS) in the South-West of the Netherlands were screened for ASD with the parent-reported Social Responsiveness Scale (SRS; Constantino and Gruber 2012). Children were referred to these centers for all kinds of developmental, emotional, and behavioral problems. The screening phase of the included children took place between April 2011 and July 2012. The local medical ethics committee and participating mental health care centers approved this study (MEC-2011-078) before the start of the data collection. We obtained written informed consent for all assessments in all phases from all individual participants included in the study.

**Fig. 1:**
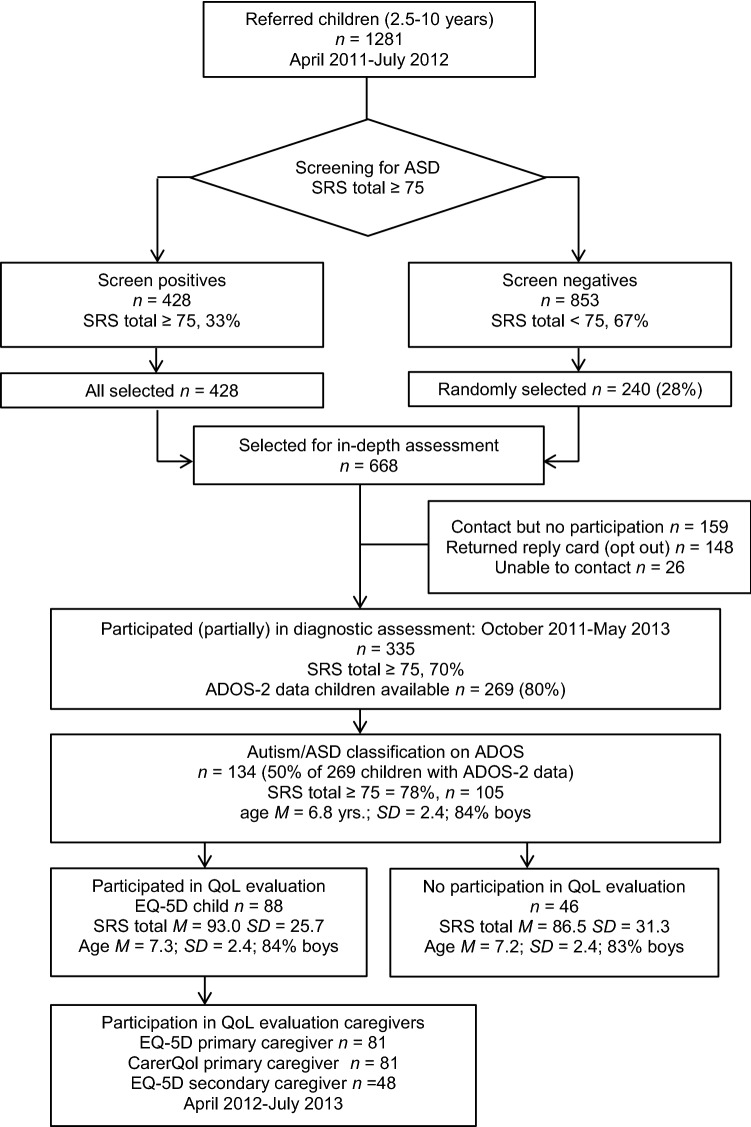
Flowchart of the study sample

Children with a high risk of ASD were selected by using a total raw score of 75 or more on the SRS as the cut-off value, thus differentiating between high risk of ASD versus other psychiatric problems with a sensitivity of 0.85 and a specificity of 0.75 (Constantino and Gruber 2005). Besides all children with a positive screening result based on the SRS (total raw score ≥ 75; *n* = 428), a random selection of children with a negative screening result based on the SRS (total raw score < 75; *n *= 240), were invited with their caregivers for an in-depth assessment (total *n* = 668), using an oversampling design (Dunn et al. 1999; Duvekot et al. 2017). We measured autistic traits in the children with the Autism Diagnostic Observation Schedule, second edition (ADOS-2, Lord et al. 2012; De Bildt et al. 2013). The assessment also included child emotional and behavioral problems, child and caregiver HRQoL, and CarerQol. The participating caregivers were the most involved *informal* caregivers, who predominantly raised and cared for the child. Caregivers reported whether they were the primary or secondary caregiver, as well as their relationship with the child (biological parent, foster parent, adoption parent, grandparent, step-parent, or otherwise). Formal caregivers, such as physicians, psychologists, or social workers, were not included in the study. The procedure, instruments, and questionnaires were identical for the participating screen-positive children and their families as well as for the screen-negative children and their families in the sample. The flowchart of the study (Fig. 1) shows that the final study sample consisted of 88 children with a classification ‘Autism’ or ‘Autism Spectrum Disorder’ on ADOS-2 data *and* available information about their HRQoL (of which 72 children with a total SRS raw score ≥ 75). Because no HRQoL questionnaire was available for 46 (134 minus 88) children with an ADOS ASD classification, they were excluded from the current study.

### Measures

The SRS (Constantino and Gruber 2005, 2012; Roeyers et al. 2011; Duvekot et al. 2015b) was used to assess the ASD risk of the children (*n* = 88). The questionnaire was completed by the primary caregiver during the period of April 2011 till July 2012. Because of the age range, the versions for preschool children, aged 2.5 to 4 years (*n* = 12; 14%), and school-age children, aged 4 years and above (*n* = 76; 86%), were used. These versions were largely similar, with only a few age-appropriate item content differences. All these children were classified with ‘Autism’ or ‘Autism Spectrum Disorder’ on the Autism Diagnostic Observation Schedule, second edition (ADOS-2; Lord et al. 2012; De Bildt et al. 2013). Fully trained and certified professionals conducted this semi-structured, standardized observation of the child’s social communication and behavior during the period of October 2011 till May 2013. Emotional and behavioral problems of the children were scored on the Child Behavior Checklist (CBCL; Achenbach and Rescorla 2000, 2001) by the primary caregiver (*n* = 81) during the screening phase, from April 2011 till July 2012, to assess levels of internalizing and externalizing problem behavior on a 3-level answering scale. Specific versions were used for children aged 1.5–5 years (*n* = 35; 43%), and for children aged 6–18 years (*n* = 46; 57%). Because of the use of two age-dependent versions, we used *t* scores to ensure the comparability of scores. Previous research confirmed the good psychometric properties of the CBCL in ASD samples (Pandolfi et al. 2009, 2014).

### Health-Related Quality of Life

The impact of ASD of the child on their HRQoL (proxy report of the primary caregiver) and that of their primary and secondary caregivers (both self-report) was measured during the period of April 2012 till July 2013 with the EuroQol Five Domain Health Questionnaire (EQ-5D, Brooks 1996; The EuroQol Group 1990; www.euroqol.org). The five domains of the EQ-5D (mobility, self-care, usual activities, pain/discomfort, and anxiety/depression) were scored using three levels: no problems (1), some problems (2), or a lot of problems (3). Each unique combination of five scores represented a different health state, which was previously valued by general public preferences to be in this specific health state. We calculated the so-called ‘EQ-5D utility scores’ using these Dutch tariffs (Lamers et al. 2006) for the observed health states. Utility scores range between 0 (reflects the score of a state equal to being dead) and 1 (reflects the score of a state of perfect health), although negative utility scores are also possible for states considered to be worse than death. Health was also directly assessed using a visual analog scale (EQ-VAS) ranging from 0 (worst imaginable health state) to 100 (best imaginable health state), which is a standard part of the EQ-5D instrument. Health utility scores of children, and both caregivers were compared with Dutch general population norms (Stolk et al. 2009). Bouwmans et al. (2014) found the EQ-5D with three-level answers to be valid for proxy measuring HRQoL in children with other developmental disorders, e.g., attention-deficit/hyperactivity disorder (ADHD). The performance of the EQ-5D with the three-level answers is fair in caregivers of children with ASD, with good validity and adequate item-total correlations, but a somewhat lower internal consistency (Cronbach’s α = 0.63; Khanna et al. 2013a).

### Care-Related Quality of Life

The impact of caring for a child with ASD on the primary caregiver was measured with the CarerQol Instrument (Brouwer et al. 2006) during the period of April 2012 till July 2013. All five negative care-related dimensions (*relational problems* with the care recipient, *mental health problems*, *problems combining the care* with daily activities, *financial problems* because of the care tasks, and *physical health problems*) and both positive care-related dimensions (*fulfillment* with carry out the care tasks and *support* with informal care tasks from family, friends, neighbors, acquaintances, when needed) of the CarerQoL were scored at three answering levels: no (1), some (2), or a lot of (3) problems/support/fulfilment. Each unique combination of seven scores represents a different caring state, which was previously valued by a sample from the Dutch general population (Hoefman et al. 2011, 2014a). Utilities, calculated by using this value set, could range from 0 (worst caring situation) to 100 (best caring situation). Additionally, the caregiver happiness was rated on a VAS (Brouwer et al. 2006) ranging from completely unhappy (0) to completely happy (10), as a standard part of the CarerQol. Previous research suggested the CarerQol is suitable to measure the impact of caring for children with developmental problems, including ASD (Payakachat et al. 2011; Hoefman et al. 2014b).

### Statistical Analyses

To determine potential selective attrition within the entire group of 134 children with an autism or ASD classification on the ADOS, we tested possible differences between the group of 88 children with a completed EQ-5D versus the group of 46 children (134 minus 88) without a completed EQ-5D with *t* and Mann–Whitney tests.

To address the aims of this study, we analyzed data of the study sample of 88 children with an autism or ASD classification on the ADOS *and* a completed EQ-5D. For these 88 children, data of 81 primary caregivers and 48 secondary caregivers were available. First, to provide insight into the HRQoL of the children and both caregivers, frequencies (answering levels on EQ-5D), means and standard deviations (EQ-5D utility and EQ-VAS scores) were calculated. When discussing the overall level of problems on an EQ-5D domain, we combined the answering levels ‘some problems’ (2) and ‘a lot of problems’ (3) into one category, leaving the answering level ‘no problems’ (1) as the other category. We used one-sample *t*-tests in a sex- and age-weighted comparison between the EQ-5D utility and EQ-VAS scores of the children, primary, and secondary caregivers and the general Dutch population norm scores (Stolk et al. 2009). We investigated the relationships between the EQ-5D utility and EQ-VAS scores of children and both caregivers by using Spearman’s rank correlation coefficients. Second, to assess the caring impact, frequencies (answering levels of CarerQol), means, and standard deviations (utility and happiness scores) of the primary caregivers, were reported. When discussing the overall level of problems on a CarerQol dimension, we combined answering levels ‘some’ (2) and ‘a lot’ (3) into one category, with the answering level ‘no’ (1) as the other category. Third, we studied the relationships between the EQ-5D domain, utility and VAS scores, for the children and each caregiver with the CarerQol dimensions and utility scores of the primary caregivers, calculating Spearman’s rank correlation coefficients. Because of multiple testing, we used an *α*-level of 0.01 to indicate significant results. We used SPSS version 24.0 to perform the analyses.

## Results

### Sample Characteristics of Children with ASD and Caregivers

No significant differences were found between our study sample of 88 ASD diagnosed children *with* a completed EQ-5D instrument and those 46 *without* a completed EQ-5D, with regard to age (*p* = 0.833), sex (*p* = 0.827), CBCL internalizing problems (*p* = 0.649), and CBCL externalizing problems (*p* = 0.248), SRS total score (*p* = 0.197), and child ethnicity (*p* = 0.317). However, children with an EQ-5D report were more likely to live with both biological parents (*p* = 0.016), as well as (relatedly) with both caregivers (*p* = 0.003).

The characteristics of the 88 participating children and their caregivers are presented in Table 1. The children had an average age of 7.3 years (*SD* = 2.4), with 84% being boys. Most children were of Dutch ethnicity (78%) and lived with both biological parents (76%). The mean total IQ of the children was 94.3 (*SD* = 18.6). The mean total raw score on the parent-reported SRS was 93.0 (*SD* = 25.7), with an ADOS-2 classification of ‘Autism’ in 64% of the children. On the CBCL, caregivers reported that 67% of the children had internalizing problems in the clinical range and 60% of the children had externalizing problems in the clinical range. Next to the proxy reports for the children, self-reports of the EQ-5D and CarerQol of primary caregivers (*n* = 81; 90% female; 89% biological mother of the child) and self-reports of the EQ-5D of secondary caregivers (*n* = 48; 88% male; 77% biological father of the child) were available.

**Table 1:** Participant characteristics of children and both caregivers with a completed EQ-5D

	Children (*n* = 88)	P1 (*n* = 81)	P2 (*n *= 48)
	*M* (*SD*)/*n* (%)	*M* (*SD*)/*n* (%)	*M* (*SD*)/*n* (%)
Male gender: *n* (%)	74 (84)	8 (10)	42 (88)
Age at assessment in years: *M* (*SD*)	7.3 (2.4)	37.2 (5.2)	42.7 (6.8)
Dutch ethnicity^a^: *n* (%)	69 (78)	61 (75)	37 (77)
High urbanicity^b^: *n* (%)	52 (59)	–	–
Children			
Full-scale IQ^c^: *M* (*SD*)	94.3 (18.6)	–	–
CBCL problems in the clinical range			
Internalizing total *t* score, *n* (%)	59 (67)		
Externalizing total *t* score, *n* (%)	53 (60)		
SRS parent report total score: *M* (*SD*)	93.0 (25.7)	–	–
SRS parent report total score ≥ 75: *n* (%)	72 (82)	–	–
ADOS total score (all modules): *M* (*SD*)	11.2 (3.7)	–	–
ADOS calibrated severity score: *M* (*SD*)	6.3 (1.8)	–	–
ADOS classification autism: *n* (%)	56 (64)	–	–
Birth order: first born child: *n* (%)	48 (55)	–	–
Living with both caregivers: *n* (%)	76 (86)	–	–
Living with both biological parents: *n* (%)	67 (76)	–	–
Caregivers			
Biological parent: *n* (%)	–	79 (98)	42 (88)
Biological mother: *n* (%)	–	72 (89)	–
Biological father: *n* (%)	–	–	37 (77)
Parental education high^d^: *n* (%)	–	21 (26)	11 (23)
Paid employment: *n* (%)	–	61 (75)	44 (92)

*ASD* autism spectrum disorders, *ADOS* Autism Diagnostic Observation Schedule, *CBCL* Child Behavior Checklist, *EQ*-*5D* EuroQol Five Domain Health Questionnaire, *SRS* Social Responsiveness Scale, *P1* primary caregiver, most involved in caring for the child, *P2* secondary caregiver, second-most involved in caring for the same child

^a^Ethnicity was classified as Dutch if both parents were born in the Netherlands

^b^Urbanicity is high with ≥ 1500 addresses per square kilometer

^c^Total IQ scores were obtained from the patient file if the IQ assessment had been conducted within the past 2 years. Most used IQ tests were the various Wechsler Intelligence Scales, third Dutch editions. IQ assessment was conducted by trained members of the research team if no recent or valid IQ assessment was available. IQ or developmental scores were missing in eight children

^d^Parental education was categorized as high with higher vocational education or university education as the highest level of completed education

### Health-Related Quality of Life Reports for Children with ASD and Both Caregivers

#### EQ-5D Domain Scores

Table 2 presents the distribution of the problem scores on the five EQ-5D domains of all 88 children, 81 primary caregivers, and 48 secondary caregivers. For the children, most problems were reported on domains ‘usual activities’ (*n* = 67; 76%) and ‘self-care’ (*n* = 53; 60%). The highest percentage of serious (‘a lot’) problems was in the ‘self-care’ domain (18%) as well. Almost half of the primary caregivers (48%) stated that their children experienced some or a lot of problems in the anxiety/depression domain. In line with their age, and perhaps also due to the nature of ASD, the least problems were reported in the domains ‘pain/discomfort’ (27%) and ‘mobility’ (18%).

**Table 2:** Frequency and percentage distribution of participants’ HRQoL responses on the five items of the EuroQol Five-Domain Questionnaire (EQ-5D)

Participants	*n*	Responses on EQ-5D items				
		Mobility, *n* (%)	Self-care, *n* (%)	Usual activities, *n* (%)	Pain/discomfort, *n* (%)	Anxiety/depression, *n* (%)
Children	88					
No problems		72 (81.8)	35 (39.8)	21 (23.9)	64 (72.7)	46 (52.3)
Some problems		15 (17.0)	37 (42.0)	55 (62.5)	20 (22.7)	36 (40.9)
A lot of problems		1 (1.1)	16 (18.2)	12 (13.6)	4 (4.5)	6 (6.8)
Primary caregivers	81					
No problems		72 (88.9)	79 (97.5)	64 (79.0)	47 (58.0)	49 (60.5)
Some problems		9 (11.1)	2 (2.5)	17 (21.0)	32 (39.5)	30 (37.0)
A lot of problems		0 (0.0)	0 (0.0)	0 (0.0)	2 (2.5)	2 (2.5)
Secondary caregivers	48					
No problems		46 (95.8)	48 (100)	44 (91.7)	41 (85.4)	41 (85.4)
Some problems		1 (2.1)	0 (0.0)	4 (8.3)	6 (12.5)	7 (14.6)
A lot of problems		1 (2.1)	0 (0.0)	0 (0.0)	1 (2.1)	0 (0.0)

The 81 primary caregivers (mostly the mothers) reported most problems in the domains ‘pain/discomfort’ (42%) and ‘anxiety/depression’ (40%) of the EQ-5D. Some, but no serious problems were reported in the domains ‘usual activities’ (21%), ‘mobility’ (11%), and ‘self-care’ (3%). Overall, the 48 secondary caregivers in our sample (mostly the fathers) reported much fewer health problems than the primary caregivers on the EQ-5D, but with a similar distribution pattern. Most problems were indicated in the domains ‘pain/discomfort’ and ‘anxiety/depression’ (both 15%, but in different caregivers). Least problems were reported in the domains ‘usual activities’ (8%) and ‘mobility’ (4%). No problems were reported in the domain of ‘self-care’.

Also, the EQ-5D scores and problem distributions of the primary and secondary caregivers of the same children were compared (*n* = 45). All responses differed to some extent, but not substantially (data not presented). We found reasonably comparable score distributions for these children and caregivers, with the most problems reported on the same domains, when compared to the full sample.

#### EQ-5D Scores Compared to Dutch Population Norm Scores

Table 3 presents the distributional characteristics of the EQ-5D utility and VAS scores of the children and both caregivers. We performed sex- and age-weighted comparisons between the health utility and VAS scores of the participants and the mean Dutch general population norm scores (Stolk et al. 2009). The mean EQ-5D utility score of the children in our study sample was 0.67 (*SD* = 0.26), indicating a significantly lower HRQoL compared to the mean Dutch norm score (0.94; *p *< 0.001). Their mean EQ-VAS score was slightly, but not significantly, lower than the mean Dutch norm score (84.97 [*SD* = 15.17] vs. 86.19). The mean EQ-5D utility score in the 25% of children with the most comorbid problems (as reported on the CBCL) decreased significantly to 0.62 (*SD* = 0.34, *p* = 0.011), compared to 0.67 in the full sample, and a mean EQ-VAS score of 82.82 (*SD* = 21.79).

**Table 3:** Distributional characteristics of EuroQol Five-Domain Questionnaire (EQ-5D): mean, median, ranges, the *p* value of one-sample *t*-tests between the sample mean and Dutch norm value, weighed for sex and age

EQ-5D scales	*n*	Mean (*SD*)	Median (range)	Norm value mean (*SD*)	*p*-value*
Children					
EQ-5D utility score	88	0.67 (0.26)	0.75 (− 0.20 to 1.00)	0.94 (0.07)	< 0.001*
EQ-VAS score	88	84.79 (15.17)	90 (10 to 100)	86.19 (3.53)	0.451
Primary caregivers					
EQ-5D utility score	81	0.84 (0.17)	0.84 (0.24 to 1.00)	0.87 (0.18)	0.089
EQ-VAS score	71	79.96 (12.67)	81 (50 to 100)	76.81 (15.02)	0.040
Secondary caregivers					
EQ-5D utility score	48	0.93 (0.14)	1.00 (0.33 to 1.00)	0.88 (0.18)	0.029
EQ-VAS score	22	82.08 (19.78)	80 (0 to 100)	78.33 (14.44)	0.362

*VAS* visual analog scale, *EQ-VAS* current health state (0–100.00, with 100.00 being completely healthy)

*Statistically significant differences (*p* < 0.01)

Mean EQ-5D utility and VAS scores of the primary and secondary caregivers were not significantly different from the respective mean Dutch norm scores, although Table 3 shows that mean EQ-5D utility scores of primary caregivers were lower, and mean EQ-5D-utility scores of secondary caregivers, as well as EQ-VAS scores of both caregivers, were somewhat higher than their counterparts in the general Dutch population. The primary caregivers of the subsample children with the most comorbid problems reported lower HRQoL with an EQ-5D utility score of 0.80 (*SD* = 0.21) and EQ-VAS score of 74.00 (*SD* = 14.78). These scores still were not significantly lower than the mean (age- and sex-adjusted) Dutch norm scores.

#### Relationships Between EQ-5D Scores of All Participant Groups

Investigating possible relationships between EQ-5D utility and EQ-VAS scores of the children, primary and secondary caregivers, we used Spearman’s rank correlation coefficients. Positive correlations between EQ-5D utility and EQ-VAS scores of the children were found (*r* = 0.439, *p* < 0.01). No significant correlations were found between the EQ-5D and VAS scores of the children and those of both caregivers, nor between those of primary and secondary caregivers.

### Care-Related Quality of Life in Primary Caregivers

**Fig. 2:**
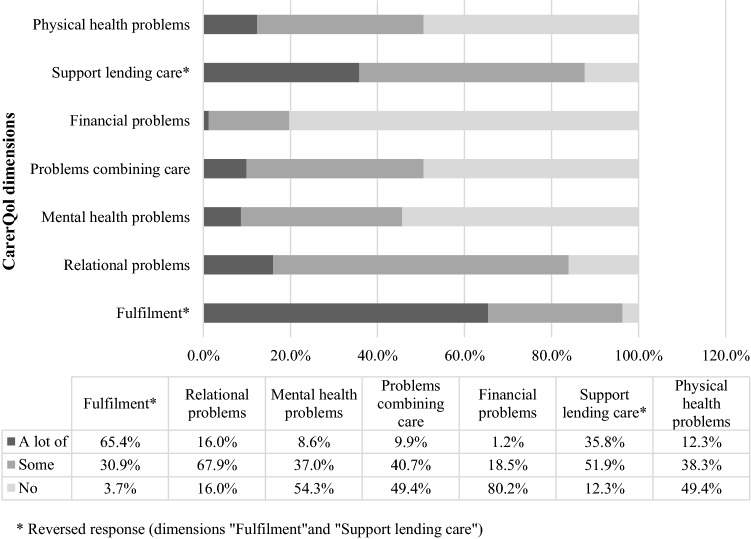
Distribution of CarerQol reports among primary caregivers (*n* = 81)

Figure 2 presents the CarerQol scores of primary caregivers, with the frequencies of the scores on the seven CarerQol dimensions (two positive and five negative ones). Most (some or a lot of) problems were reported on the dimension ‘relational problems with the care recipient’ (84%). Next to this, about half of the caregivers indicated at least some problems on the dimensions ‘combining the care with daily activities’ (51%), ‘physical health problems’ (51%), and ‘mental health problems’ (46%). Financial problems were reported by 20% of the caregivers. Notwithstanding these scores, nearly all primary caregivers reported deriving (some or a lot of) fulfillment from caring for their children (96%, of whom 65% ‘a lot’) and experiencing support in providing their care (88%, of whom 36% ‘a lot’). Table 4 presents distributional characteristics of the CarerQol scores, with a mean utility score of 77.33 and an average happiness score of 7.6 (CarerQol-VAS) in primary caregivers. The mean utility and happiness scores of the 45 primary caregivers with secondary caregivers in the study sample (caregivers of the same children) did not differ from the full sample. Also, the same distribution of CarerQol scores on the different dimensions was observed in this subsample (data not presented).

**Table 4:** Distributional characteristics of the Care-Related Quality of Life Questionnaire (CarerQol) of primary caregivers

CarerQol scales	*n*	Mean	Median	SD	Range
CarerQol utility score	76	77.33	80.55	16.44	17.00–98.00
CarerQol-VAS	71	7.56	7.60	1.42	2.3–10.0

*VAS* visual analog scale, *CarerQol-VAS* happiness score primary caregiver (0–10.0, with 10.0 being completely happy)

### Relationships Between Health- and Care-Related Quality of Life

Table 5 presents the Spearman’s correlation coefficients between the CarerQol dimensions and utility scores of the primary caregivers and their EQ-5D utility and VAS scores, as well as those of the children. CarerQol utility scores of the primary caregivers were significantly positively correlated with their EQ-5D utility scores (*p* < 0.001), as well as with EQ-5D utility scores of their children (*p* = 0.002). Higher CarerQol scores thus appeared to be associated with higher EQ-5D scores of the primary caregivers and higher EQ-5D scores of their children. Also, the happiness of the primary caregiver (CarerQol-VAS) was significantly correlated with EQ-VAS scores of caregivers (*p* < 0.001, not presented in the table). Also, the same correlations were found in the selection of primary and secondary caregivers of the same children (*n* = 45).

**Table 5:** Spearman’s correlation coefficients of children’s and primary caregivers’ responses on the EuroQol Five-Dimension Questionnaire (EQ-5D item and EQ-5D utility scores, including VAS) with the primary caregivers’ responses on the Care-Related Quality of Life Questionnaire (CarerQol item and CarerQol utility scores)

EQ-5D domains	CarerQol-7D dimensions							
	Fulfillment	Relational problems	Mental health problems	Problems combining daily act	Financial problems	Support lending care	Physical health problems	CarerQol utility score
Child								
Mobility	0.067	0.173	0.175	0.103	0.178	0.043	0.168	− 0.074
Self-care	− 0.019	0.300*	0.139	0.278	0.138	− 0.061	0.186	− 0.236
Usual activities	− 0.141	0.403*	0.059	0.362*	0.256	− 0.057	0.087	− 0.163
Pain/discomfort	− 0.133	0.206	0.147	0.288*	0.007	− 0.251	0.043	− 0.198
Anxiety/depression	− 0.291*	0.295*	0.268	0.355*	0.027	− 0.062	0.230	− 0.325*
EQ-5D utility score	0.145	− 0.351*	− 0.274	− 0.456*	− 0.142	0.055	− 0.276	0.345*
EQ-VAS	0.127	− 0.198	− 0.128	− 0.373*	− 0.033	0.049	− 0.081	0.181
Primary caregiver								
Mobility	0.018	0.208	0.000	0.073	0.019	− 0.142	0.157	− 0.118
Self-care	− 0.045	0.140	0.000	0.198	− 0.079	− 0.072	0.113	− 0.101
Usual activities	− 0.198	0.161	0.378*	0.299*	0.135	− 0.127	0.578*	− 0.540*
Pain/discomfort	− 0.045	0.134	0.151	0.159	0.181	− 0.076	0.551*	− 0.296*
Anxiety/depression	− 0.196	0.173	0.615*	0.276	0.099	− 0.295*	0.439*	− 0.461*
EQ-5D utility score	0.198	− 0.211	− 0.532*	− 0.288*	− 0.118	0.270	− 0.657*	0.551*
EQ-VAS	0.097	− 0.084	− 0.171	− 0.225	0.052	− 0.002	− 0.060	0.143

*VAS* visual analog scale, *EQ-VAS* current health state (0–100.00, with 100.00 being completely healthy), *CarerQol-VAS* happiness score primary caregiver (0–10.0, with 10.0 being completely happy)

*Statistically significant differences (*p* < 0.01)

Lower CarerQol scores of primary caregivers were significantly correlated with more anxiety/depression (*p* = 0.004) of their children on the EQ-5D. Moreover, these were correlated with more self-reported anxiety/depression (*p* < 0.001), more self-reported problems with usual activities (*p* < 0.001), and more self-reported pain/discomfort (*p* = 0.009) of the primary caregivers on the EQ-5D (Table 5). Thus, lower CarerQol scores of primary caregivers were associated with higher anxiety/depression in their children and themselves. Also, their care-related QoL was lower with more own pain/discomfort and problems with usual activities.

More problems of caregivers in the relationship with their child, as indicated on the CarerQol, were significantly correlated with more child problems in self-care (*p* = 0.007), usual activities (*p* < 0.001) and with anxiety/depression (*p* = 0.007), as indicated on the EQ-5D. More problems of caregivers in the CarerQol dimension of combining daily activities and care were significantly correlated with more child problems on the EQ-5D domains usual activities (*p* = 0.001), pain/discomfort (*p* = 0.009), and anxiety/depression (*p* = 0.001), as well as with own problems in the EQ-5D domain usual activities (*p* = 0.007).

Less fulfillment from caring of primary caregivers, as indicated on the CarerQol, was significantly correlated with more anxiety/depression problems in children (*p* = 0.008) as measured with the EQ-5D. Less support in caring for their child, indicated on the CarerQol was correlated with more caregiver anxiety/depression (*p* = 0.007) as measured with the EQ-5D. More mental health problems of caregivers, as reported on the CarerQol, were significantly correlated with more problems on the EQ-5D domains usual activities and anxiety/depression in caregivers (*p* < 0.001). More physical health problems of caregivers, as indicated on the CarerQol, were significantly correlated with EQ-5D domains usual activities, pain/discomfort, and anxiety/depression of the caregivers (*p* < 0.001).

No statistically significant correlations were found between any of the CarerQol dimensions, utility and VAS scores of primary caregivers and the EQ-5D domain, utility and VAS scores of secondary caregivers (data not presented).

## Discussion

This study was the first one to investigate HRQoL of children, aged 2–10 years, with an ADOS ASD classification and that of both their caregivers (primary and secondary), as well as care-related QoL of their primary caregivers.

Compared with Dutch norm scores, the HRQoL of the children in this study proved relevantly reduced with an average EQ-5D utility score of 0.67. The average EQ-5D utility score decreased even further to 0.62 in the 25% of children with most comorbid problems on the CBCL. Most problems in the whole group of children were reported in the domains self-care, usual activities, and anxiety/depression; the same problem distribution on the EQ-5D was observed in the 25% of children with most comorbid problems on the CBCL. For primary and secondary caregivers, no lowered HRQoL was found. Caregivers reported most problems in EQ-5D domains anxiety/depression and pain/discomfort, primary caregivers more than secondary caregivers. In the context of caring, most primary caregivers indicated problems in the relationship with the child they cared for, and difficulties in combining care with daily activities. Notwithstanding these problems, the majority of the primary caregivers also reported to experience fulfillment from and support with care. Care-related QoL and HRQoL of primary caregivers were correlated, as were care-related QoL of primary caregivers and HRQoL of their children. We found no correlations with any of the HRQoL scores of the secondary caregiver. About the same findings and correlations were found in the selection of primary and secondary caregivers of the same children.

Reduced HRQoL in children with ASD was also observed in previous studies with other instruments (Bastiaansen et al. 2004; Kuhlthau et al. 2013). In our study, the domains ‘usual activities’ and ‘self-care’ were primarily affected by ASD. Because the EQ-5D utility scores were not reported in previous ASD studies, direct comparisons were impossible. EQ-5D utility scores of the children in our study were lower than those previously reported for children with other developmental disorders, such as ADHDs with reported EQ-5D utility scores of 0.80 (Van der Kolk et al. 2014) and 0.75 (Matza et al. 2005) or with somatic conditions, such as an imperforate anus (0.87; Stolk et al. 2000) or a congenital diaphragmatic hernia (0.92; Poley et al. 2002).

The finding of no significantly lowered HRQoL in primary caregivers of children with ASD contrasts with the comparisons to general population norms in previous studies (Mugno et al. 2007; Khanna et al. 2013b). Kuhlthau et al. (2014) found a significantly lower HRQoL compared to the general US population in caregivers of ASD children with the use of the Six Dimension Short-Form Health Survey (SF-6D), but not with the use of the EQ-5D. Thus, differences may be partially explained by the use of different QoL questionnaires, which emphasizes the importance of choice of tools. Differences may also relate to sample characteristics. In our sample, no adolescents were included, and the children were somewhat less impaired concerning autism and intellectual level than in some previous studies. The characteristics of caregivers were comparable to those of other studies (e.g., age, marital status, education, employment), although information about household income lacked in our study. EQ-5D utility scores of primary caregivers were comparable to those of Kuhlthau et al. (2014).

We also found no statistically significant lowered HRQoL of secondary caregivers, compared to Dutch population norm scores. Our results for secondary caregivers are novel and hence can not be compared to previous findings. No significant, but somewhat higher EQ-5D utility scores of the secondary caregiver and higher EQ-VAS scores of both caregivers were found, compared with Dutch population norms (Stolk et al. 2009). This could be due to a possible selection bias of somewhat more healthy caregivers participating in our study. The lower HRQoL of primary caregivers (of whom 90% female) compared to secondary caregivers (of whom 88% male) in our study is in line with general population norms with lower HRQoL of females compared with males (Stolk et al. 2009). Also, this is consistent with other ASD studies, reporting lower HRQoL in female caregivers with more stress, anxiety, and depression than in male caregivers (Allik et al. 2006; Khanna et al. 2013b).

Concerning the impact of ASD on caring, we found a much higher proportion of caregivers reporting relational problems with the child compared to the US study by Hoefman et al. (2014b) on the CarerQoL (84% vs. 45%). This remarkable difference did not seem to be explained by differences in the sample. Possibly, Dutch attitudes toward caring for a child with the challenges due to ASD, as well as Dutch opinions about parent–child interaction in child-raising, are responsible for this difference in response to US caregivers. Speculatively, one might expect Dutch caregivers to be less reluctant and ashamed of reporting perceived relational problems with their child in comparison to American caregivers, but no ASD study has focused on this topic to our knowledge. Financial problems were less reported on the CarerQol in our study compared to the US situation (20% vs. 56%, Hoefman et al. 2014b), presumably because of the Dutch health care reimbursement system, as well as Dutch social security benefits, to compensate for health care expenses, sick leave or unemployment.

Consistent with other studies (Payakachat et al. 2011; Hoefman et al. 2014b; Fitzgerald et al. 2018), we found remarkably high proportions of caregivers experiencing fulfillment (96%) of and support (88%) in the care for their affected child. This important information offers an additional perspective on the impact of ASD on caregivers, with reports of both negative and positive caring aspects. Especially in parenting a child with an ASD, caregivers are continuously seeking to balance situation-specific risks and protective factors (Bonis et al. 2016). Protective factors are most likely to be found in the familial and social context, for example, support in providing the necessary care.

As expected, HRQoL of the children decreased with more comorbidity. Moreover, we found an association between more anxiety/depression in children and caregivers, and lower CarerQol scores of caregivers. Although a family risk of internalizing problems may (partly) explain this association, there was no direct correlation between child and caregiver anxiety/depression in this study. These findings are important and relevant to clinical practice. Screening and treatment of comorbid problems in children with an ASD are essential in improving the family QoL, especially concerning internalizing problems (anxiety/depression). Also, the caregivers of children with an ASD, often their parents, should be screened, and if necessary treated, for internalizing problems. Caregiver involvement is essential in the treatment of children with an ASD (Volkmar et al. 2014). The health and QoL of caregivers are often neglected in the process of (evaluation of) child treatment, but the results of this study stress the need for more attention paid to these aspects of caregiving. Improving the health and QoL of caregivers is even more critical because of the observed interaction between the QoL of the child and that of the primary caregiver.

These positive correlations between CarerQol and HRQoL of primary caregivers as well as HRQoL of children may be explained by family transactional effects with a continuous reciprocal interaction process between children and their caregivers (Rodriguez et al. 2019). Also, spillover effects may explain positive correlations between QoL of children and of caregivers (Bobinac et al. 2010, 2011; Wittenberg et al. 2013), with the family effect (i.e., the QoL of family members, including the caregivers, is influenced by the caring about the child’s health) and the caregiving effect (i.e., the QoL of the caregiver is associated with the burden of caring for the child). Moreover, the fact that the same (primary) caregiver reported all these QoL aspects in the self-report and proxy report, and the influence of endogeneity with possible shared genetic and environmental factors of health in caregivers and children at the same time, should be taken into consideration (Duvekot et al. 2015a). The above stresses the importance of using multiple informants and perspectives in measuring health and QoL effects in children with ASD and their caregivers.

Surprisingly, we found no significant correlations between the HRQoL scores of secondary caregivers and all other HRQoL and CarerQol scores (of children and primary caregivers). Although we have to be careful drawing firm conclusions, also given the small sample size, this finding in itself could be clinically relevant, stressing the differences in time investment, perceptions, experiences, and perspectives of the caring situation between primary versus secondary caregivers that might give rise to problems in family functioning. Everhart et al. (2018), for instance, found that differences in QoL scores between both caregivers in families of children with pediatric asthma were associated with more family burden and psychological problems in the primary caregivers.

This study confirmed the previously reported finding that children with an ASD have a significantly lower HRQoL. A lower HRQoL was not observed in primary and secondary caregivers in our study. In the group of children with the most comorbid problems, we found an even lower HRQoL of the children and their primary caregivers. The absence of significant correlations between HRQoL of the children and their caregivers, and between the HRQoL of both caregivers, suggest that these reports each provide unique information about the impact of a child with an ASD in the family. The correlations of the CarerQol with some HRQoL aspects of the children and caregivers themselves may be (partly) explained by family transactions and spillover effects. Despite the serious problem scores, the majority of caregivers also experience positive aspects of caring for their child, which may be a reflection of resilience in caregivers but also their desire to care for their child and the feelings of the reward of doing so. These findings have implications for outcome measurement and interventions in the context of children with an ASD, but before highlighting these, we first discuss some strengths and limitations of this study.

### Strengths and Limitations

One strength of the study we want to emphasize, was the use of self-reports of both primary and secondary caregivers, next to proxy reports of children with an ASD to provide new insights in correlations between the HRQoL of all participants. Second, both HRQoL and CarerQol of primary caregivers were measured to capture different aspects of caring for children with ASD. Importantly, high scores on positive aspects of caring were reported by the caregivers, which cannot be found using HRQoL reports.

This study also had some noteworthy limitations. First, this Dutch multicenter study included a well-defined and relevant sample (Duvekot et al. 2017) but was relatively small compared to most US studies (Hoefman et al. 2014b; Kuhlthau et al. 2014). Our participants may not be fully representative of the total group of children with ASD and their caregivers, because relatively many children lived with both biological parents, were somewhat younger, and appeared to be slightly less impaired intellectually and with regard to autism traits (Table 1), compared to those in other study samples. Despite this possible bias, we still found severe impairments in HRQoL in children. Second, no self-report of the children with ASD was available. Children with ASD tend to report their own QoL somewhat higher than their parents (Sheldrick et al. 2012; Clark et al. 2014). Proxy-reported QoL of children should only be used when children are too young, too ill, or too disabled to self-report (Coghill et al. 2009). Despite an available child-friendly form of the EQ-5D for the age group 8–16 years (EQ-5D-Y, Wille et al. 2010), we preferred the parent-report, because of the age range in our study (2–10 years), the use of one version of a HRQoL instrument in the whole sample, and the presence of a disorder in the children. We also note that the fact that primary caregivers not only self-reported their health and QoL but also proxy-reported that of the child may have influenced our results. In particular, the correlations between the HRQoL in children and primary caregivers may be affected by the fact that they stemmed from the same source. Third, Dutch population scores for the EQ-5D for children (Stolk et al. 2009) were based on a rather small sample with a different age range (5–14 years). No other Dutch population norms for children in the appropriate age range or norm scores for children with ASD were available. Fourth, although the EQ-5D had advantages like feasibility, applicability in a broad age range, and the possibility to calculate preference-based utilities, it also has limitations. Although there is some debate on the appropriateness of HRQoL measurements, the use of the EQ-5D in the assessment of the perceived health state seems appropriate (Karimi and Brazier 2016). Also, one may question whether such a generic HRQoL instrument is sensitive enough to be used in children with autism and their caregivers. However, Khanna et al. (2013a) demonstrated adequate psychometric properties of the EQ-5D in caregivers of children with ASD. Fifth, secondary caregivers did not complete the CarerQol instrument. Nonetheless, based on differences in HRQoL between both caregivers in our study sample, and based on the study results using the CarerQol in both caregivers of children with cystic fibrosis (Fitzgerald et al. 2018), assessment of the caregiver burden as perceived by both caregivers could indeed be valuable.

### Clinical Implications and Further Research

The HRQoL and care-related QoL instruments, as used in this study, provide options for outcomes assessments, intervention studies, and economic evaluations in families with ASD children for individual patient care, but also health care decision-making and policy. Each instrument provides a unique addition to measurements. Because of the entanglement between the child’s QoL and caregivers’ QoL, both perspectives should be included in the ASD clinical assessment.

Although caregivers reported a lot of fulfillment and informal support in the caregiving, also many problems were reported on the child and caregiver level. The findings of our study suggest ways to improve family QoL, with interventions focusing on increasing self-care skills and diminishing anxiety/depression problems in the children. Also, preventing or reducing physical and mental health problems of primary caregivers, as well as improving their broader wellbeing, for instance by facilitating combining care tasks with daily activities, will directly contribute to the family QoL.

Further research of HRQoL and care-related QoL in more extensive, representative samples of children with ASD, as well as in children with other developmental disorders, and all their caregivers are needed to be able to generalize and compare these results. Such studies can also shed more light on some issues that were underexplored in this study. For instance, HRQoL and care-related QoL of secondary caregivers, also in relation to that of primary caregivers, as well as to child, caregiver and caregiving situation characteristics should be further examined. The finding of the even lower HRQoL in children with the most comorbid problems is especially relevant for this recommendation. This study indicates that child and caregiver outcomes are associated. Next to focusing on the child characteristics influencing QoL of children and their caregivers, also caregiver characteristics should be included (Allik et al. 2006; Kuhlthau et al. 2014). For intervening in ASD, it is relevant to know which factors would have the highest probability of improving family QoL.

## Conclusion

In this study, relevantly impaired HRQoL in children with ASD was found, compared to Dutch norm scores. Problems with self-care, anxiety/depression, and usual activities were most prevalent. HRQoL of primary and secondary caregivers was not lower than relevant population norms. The most prevalent problems in caregivers were pain/discomfort and anxiety/depression, although less so in secondary compared to primary caregivers. In the context of caring, problems in the relationship with the child cared for, and combining care with daily activities, were reported most often. Despite the reported problems, almost all primary caregivers stated fulfillment from and informal support in caring for their affected child. Lower care-related QoL of primary caregivers was associated with more trouble in performing usual activities, pain/discomfort, and anxiety/depression of themselves, but also with more anxiety/depression of their affected children. No significant correlations were found with any of the health-related scores of the secondary caregiver.

The absence of significant correlations between HRQoL of the children and their caregivers, and between the HRQoL of both caregivers, suggest that these reports each provide unique information about the impact of a child with an ASD in the family.

Although some child and caregiver outcomes were associated and both perspectives should be included in outcome measurement, further research is needed in more extensive, representative samples to explore these relationships, as well as the influence of child, caregiver and caregiving characteristics. This study indicates that some child and caregiver outcomes are associated. Next to focusing on the child characteristics influencing QoL of children and their caregivers, also caregiver characteristics should be included in interventions to improve the QoL of children with ASD and their families, which remains an important goal.

## References

[CR1] Achenbach TM, Rescorla LA (2000). Manual for the ASEBA preschool forms & profiles.

[CR2] Achenbach TM, Rescorla LA (2001). Manual for the ASEBA school-age forms & profiles.

[CR3] Allik H, Larsson JO, Smedje H (2006). Health-related quality of life in parents of school-age children with Asperger syndrome or high-functioning autism. Health and Quality of Life Outcomes.

[CR4] Bastiaansen D, Koot HM, Ferdinand RF, Verhulst FC (2004). Quality of life in children with psychiatric disorders: Self-, parent, and clinician report. Journal of the American Academy of Child and Adolescent Psychiatry.

[CR5] Bobinac A, Van Exel NJA, Rutten FFH, Brouwer WBF (2010). Caring for and caring about: Disentangling the caregiver effect and the family effect. Journal of Health Economics.

[CR6] Bobinac A, Van Exel NJA, Rutten FFH, Brouwer WBF (2011). Health effects in significant others: Separating family and care-giving effects. Medical Decision Making.

[CR100] Bonis SA, Sawin KJ (2016). Risks and protective factors for stress self-management in parents of children with autism spectrum disorder: An integrated review of the literature. Journal of Pediatric Nursing.

[CR7] Bouwmans C, Van der Kolk A, Oppe M, Schawo S, Stolk E, Van Agthoven M, Buitelaar J, Hakkaart-van Roijen L (2014). Validity and responsiveness of the EQ-5D and the KIDSSCREEN-1 in children with ADHD. The European Journal of Health Economics.

[CR8] Brooks R (1996). EuroQoL: The current state of play. Health Policy.

[CR9] Brouwer WB, Van Exel NJA, Tilford MJ, Ungar WJ (2009). Incorporating caregiver and family effects in economic evaluations of child health. Economic evaluation in child health.

[CR10] Brouwer WB, Van Exel NJ, Van Gorp B, Redekop WK (2006). The CarerQoL instrument: A new instrument to measure care-related quality of life of informal caregivers for use in economic evaluations. Quality of Life Research.

[CR11] Clark BG, Magill-Evans JE, Koning CJ (2014). Youth with autism spectrum disorders self- and proxy-reported quality of life and adaptive functioning. Focus on Autism and Other Developmental Disabilities.

[CR12] Coghill D, Danckaerts M, Sonuga-Barke E, Sergeant J, ADHD European Guidelines Group (2009). Practitioner Review: Quality of life in child mental health—Conceptual challenges and practical choices. The Journal of Child Psychology and Psychiatry.

[CR13] Constantino JN, Gruber CP (2005). Social Responsiveness Scale (SRS).

[CR14] Constantino JN, Gruber CP (2012). Social Responsiveness Scale, Second Edition (SRS-2).

[CR15] De Bildt, A., Greaves-Lord, K., & De Jonge, M. (2013). *ADOS*-*2: Autisme diagnostisch observatieschema. Handleiding. [ADOS 2nd edition: Autism diagnostic observation schedule. Dutch manual.]*. Amsterdam: Hogrefe.

[CR16] De Vries M, Geurts H (2015). Influence of autism traits and executive functioning on quality of life in children with an autism spectrum disorder. Journal of Autism and Developmental Disorders.

[CR17] Drummond M (2001). Introducing economic and quality of life measurements into clinical studies. Annals of Medicine.

[CR18] Dunn G, Pickles A, Tansella M, Vazquez-Barquero JL (1999). Two-phase epidemiological surveys in psychiatric research. British Journal of Psychiatry.

[CR19] Duvekot J, Ten Hoopen LW, Slappendel G, Van der Ende J, Verhulst FC, Van der Sijde A, Greaves-Lord K (2017). Design and cohort characteristics of the social spectrum study: A multicenter study of the autism spectrum among clinically referred children. Journal of Autism and Developmental Disorders.

[CR20] Duvekot J, Van der Ende J, Constantino JN, Verhulst FC, Greaves-Lord K (2015). Symptoms of autism spectrum disorder and anxiety: Shared familial transmission and cross-assortative mating. The Journal of Child and Psychological and Psychiatry.

[CR21] Duvekot J, Van der Ende J, Verhulst FC, Greaves-Lord K (2015). The Screening Accuracy of the Parent and Teacher-Reported Social Responsiveness Scale (SRS): Comparison with the 3Di and ADOS. Journal of Autism and Developmental Disorders.

[CR22] Eapen V, Guan J (2016). Parental quality of life in autism spectrum disorder: Current status and future directions. Acta Psychopathologica.

[CR23] Everhart RS, Greenlee JL, Winter MA, Fiese BH (2018). Primary and secondary caregiver report of quality of life in pediatric asthma: Are they comparable?. Applied Research in Quality of Life.

[CR24] Falk NH, Norris K, Quinn MG (2014). The factors predicting stress, anxiety, and depression in the parents of children with autism. Journal of Autism and Developmental Disorders.

[CR25] Ferrans CE, Zerwic JJ, Wilbur JE, Larson JL (2005). Conceptual model of health-related quality of life. Journal of Nursing Scholarship.

[CR26] Fitzgerald C, George S, Somerville R, Linnane B, Fitzpatrick P (2018). Caregiver burden of parents of young children with cystic fibrosis. Journal of Cystic Fibrosis.

[CR27] Gurney JG, McPheeters ML, Davis MM (2006). Parental report of health conditions and health care use among children with and without autism: National survey of children’s health. Archives of Pediatrics and Adolescent Medicine.

[CR28] Hoefman R, Payakachat N, Van Exel J, Kuhlthau K, Kovacs E, Pyne J, Tilford JM (2014). Caring for a child with autism spectrum disorder and parents’ quality of life: Application of the CarerQoL. Journal of Autism and Developmental Disorders.

[CR29] Hoefman RJ, Van Exel NJA, Redekop WK, Looren de Jong S, Brouwer WBF (2011). A new test of the validity of the CarerQoL instrument: Measuring ‘care-related quality of life’ of informal caregivers for use in economic evaluations. Quality of Life Research.

[CR30] Hoefman RJ, Van Exel J, Rose JM, Van de Wetering E, Brouwer WB (2014). A discrete choice experiment to obtain a tariff for valuing informal care situations measured with the CarerQoL instrument. Medical Decision Making.

[CR31] Karimi M, Brazier J (2016). Health, health-related quality of life, and quality of life: What is the difference?. Pharmacoeconomics.

[CR32] Karst JS, Van Hecke AV (2012). Parent and family impact of autism spectrum disorders: A review and proposed model for intervention evaluation. Clinical Child and Family Psychology Review.

[CR33] Khanna R, Jariwala K, Bentley JP (2013). Psychometric properties of the EuroQoL Five Dimensional Questionnaire (EQ-5D-3L) in caregivers of autistic children. Quality of Life Research.

[CR34] Khanna R, Jariwala K, Bentley JP (2013). Health utility assessment using EQ-5D among caregivers of children with autism. Value in Health.

[CR35] Khanna R, Madhavan SS, Smith MJ, Patrick JH, Tworek C, Becker-Cottrill B (2011). Assessment of health-related quality of life among primary caregivers of children with autism spectrum disorders. Journal of Autism and Development Disorders.

[CR36] Kheir N, Ghoneim O, Sandridge AL, Al-Ismail M, Hayder S, Al-Rawi F (2012). Quality of life of caregivers of children with autism in Qatar. Autism.

[CR37] Kuhlthau K, Kovacs E, Hall T, Clemmons T, Orlich F, Delahaye J, Sikora D (2013). Health-related quality of life for children with ASD: Associations with behavioral characteristics. Research in Autism Spectrum Disorders.

[CR38] Kuhlthau KA, McDonell E, Coury DL, Payakachat N, Macklin E (2018). Associations of quality of life with health-related characteristics among children with autism. Autism.

[CR39] Kuhlthau K, Orlich F, Hall TA, Sikora D, Kovacs EA, Delahaye J, Clemons TE (2010). Health-Related Quality of Life in children with autism spectrum disorders: Results from the autism treatment network. Journal of Autism and Developmental Disorders.

[CR40] Kuhlthau K, Payakachat N, Delahaye J, Hurson J, Pyne JM, Kovacs E, Tilford JM (2014). Quality of life for parents of children with autism spectrum disorders. Research in Autism Spectrum Disorders.

[CR41] Lamers LM, McDonnell J, Stalmeier PF, Krabbe PF, Busschbach JJ (2006). The Dutch tariff: Results and arguments for an effective design for national EQ-5D valuation studies. Health Economics.

[CR42] Lord C, Rutter M, DiLavore PC, Risi S, Gotham K, Bishop SL (2012). Autism diagnostic observation schedule, Second Edition (ADOS-2). Manual (Part I).

[CR43] Matza LS, Secnik K, Mannix S, Sallee FR (2005). Parent-proxy EQ-5D ratings of children with attention-deficit hyperactivity disorder in the US and the UK. Pharmacoeconomics.

[CR45] Mugno D, Ruta L, D’Arrigo VG, Mazzone L (2007). Impairment of quality of life in parents of children and adolescents with pervasive developmental disorder. Health and Quality of Life Outcomes.

[CR46] Pandolfi V, Magyar CI, Dill CA (2009). Confirmatory factor analysis of the child behavior checklist 1.5-5 in a sample of children with autism spectrum disorders. Journal of Autism and Developmental Disorders.

[CR47] Pandolfi V, Magyar CI, Norris M (2014). Validity study of the CBCL 6-18 for the assessment of emotional problems in youth with ASD. Journal of Mental Health Research in Intellectual Disabilities.

[CR48] Payakachat N, Tilford JM, Brouwer WB, Van Exel NJ, Grosse SD (2011). Measuring health and well-being effects in family caregivers of children with craniofacial malformations. Quality of Life Research.

[CR49] Poley MJ, Stolk EA, Tibboel D, Molenaar JC, Busschbach JJV (2002). The cost-effectiveness of treatment for congenital diaphragmatic hernia. Journal of Pediatric Surgery.

[CR50] Rodriguez G, Hartley SL, Bolt D (2019). Transactional relations between parenting stress and child autism symptoms and behavior problems. Journal of Autism and Developmental Disorders.

[CR51] Roeyers, H., Thys, M., Druart, C., De Schryver, M., & Schittekatte, M. (2011). *SRS Screeningslijst voor autismespectrumstoornissen. [Screening list of autism spectrum disorders. Manual].* Amsterdam: Hogrefe.

[CR52] Sheldrick RC, Neger EN, Shipman D, Perrin EC (2012). Quality of life of adolescents with autism spectrum disorders: Concordance among adolescents’ self-reports, parents’ reports, and parents’ proxy reports. Quality of Life Research.

[CR53] Stolk EA, Busschbach JJ, Vogels T (2000). Performance of the EuroQoL in children with imperforate anus. Quality of Life Research.

[CR54] Stolk, E., Krabbe, P., & Busschbach, J. (2009). Using the Internet to collect EQ-5D norm scores: A valid alternative? In J. Busschbach, R. Rabin & F. De Charro (Eds.), *Proceedings of the 24th Scientific Plenary Meeting of the Euro*QoL *Group* (pp. 153–165). Rotterdam: EuroQoL Executive Office. ISBN/EAN: 978-90-814425-1-0.

[CR55] The EuroQoL Group (1990). EuroQoL—A new facility for the measurement of health-related quality of life. Health Policy.

[CR56] Van der Kolk A, Bouwmans CA, Schawo SJ, Buitelaar JK, Van Agthoven M, Hakkaart-van Roijen L (2014). Association between quality of life and treatment response in children with attention deficit hyperactivity disorder and their parents. Journal of Mental Health Policy and Economics.

[CR57] Versteegh MM, Brouwer WBF (2016). Patient and general public preferences for health states: A call to reconsider current guidelines. Social Science and Medicine.

[CR58] Volkmar F, Siegel M, Woodbury-Smith M, King B, McCracken J, State M, American Academy of Child and Adolescent Psychiatry (AACAP) Committee on Quality Issues (CQI) (2014). Practice parameter for the assessment and treatment of children and adolescents with autism spectrum disorder. Journal of American Academy of Child and Adolescent Academy.

[CR59] Wille N, Badin X, Bonsel G, Burstrom K, Cavrini G, Devlin N (2010). Development of the EQ-5D-Y: A child-friendly version of the EQ-5D. Quality of Life Research.

[CR60] Wittenberg E, Ritter GA, Prosser LA (2013). Evidence of spillover of illness among household members: EQ-5D scores from a US sample. Medical Decision Making.

